# Bacterial community shift in the coastal Gulf of Mexico salt-marsh sediment microcosm in vitro following exposure to the Mississippi Canyon Block 252 oil (MC252)

**DOI:** 10.1007/s13205-014-0233-x

**Published:** 2014-07-10

**Authors:** Hyunmin Koo, Nazia Mojib, Jonathan P. Huang, Rona J. Donahoe, Asim K. Bej

**Affiliations:** 1 Department of Biology, University of Alabama at Birmingham, 1300 University Blvd., CH464, Birmingham, AL 35294-1170 USA; 2 Department of Geological Sciences, University of Alabama, Tuscaloosa, AL 35487-0338 USA; 3 Present Address: Red Sea Research Center, King Abdullah University of Science and Technology (KAUST), Thuwal, Saudi Arabia

**Keywords:** Oil spill, QIIME, Bioinformatics, Metagenomics, Pyrosequencing, Biodegradative genes, PCR

## Abstract

In this study, we examined the responses by the indigenous bacterial communities in salt-marsh sediment microcosms in vitro following treatment with Mississippi Canyon Block 252 oil (MC252). Microcosms were constructed of sediment and seawater collected from Bayou La Batre located in coastal Alabama on the Gulf of Mexico. We used an amplicon pyrosequencing approach on microcosm sediment metagenome targeting the V3–V5 region of the 16S rRNA gene. Overall, we identified a shift in the bacterial community in three distinct groups. The first group was the early responders (orders Pseudomonadales and Oceanospirillales within class Gammaproteobacteria), which increased their relative abundance within 2 weeks and were maintained 3 weeks after oil treatment. The second group was identified as early, but transient responders (order Rhodobacterales within class Alphaproteobacteria; class Epsilonproteobacteria), which increased their population by 2 weeks, but returned to the basal level 3 weeks after oil treatment. The third group was the late responders (order Clostridiales within phylum Firmicutes; order Methylococcales within class Gammaproteobacteria; and phylum Tenericutes), which only increased 3 weeks after oil treatment. Furthermore, we identified oil-sensitive bacterial taxa (order Chromatiales within class Gammaproteobacteria; order Syntrophobacterales within class Deltaproteobacteria), which decreased in their population after 2 weeks of oil treatment. Detection of alkane (*alkB*), catechol (*C2,3DO*) and biphenyl (*bph*) biodegradation genes by PCR, particularly in oil-treated sediment metacommunity DNA, delineates proliferation of  the hydrocarbon degrading bacterial community. Overall, the indigenous bacterial communities in our salt-marsh sediment in vitro microcosm study responded rapidly and shifted towards members of the taxonomic groups that are capable of surviving in an MC252 oil-contaminated environment.

**Electronic supplementary material:**

The online version of this article (doi:10.1007/s13205-014-0233-x) contains supplementary material, which is available to authorized users.

## Introduction

The Gulf of Mexico (GoM) harbors a rich biota with ecological and high commercial significance (Yanez-Arancibia and Day 2004). In this ecosystem, the microorganisms are the primary consumers and play a major role in the biogeochemical cycling of organic matter (Pomeroy 1974). The GoM ecosystem is often perturbed by natural calamities or human activities, which affect the microbial community structure (Kryachko et al. 2012) and the associated nutrient cycling at all trophic levels (Pomeroy 1974; Camilli et al. 2010; Atlas and Hazen 2011). On April 20, 2010 the Macondo Blowout, the largest accidental oil spill in the GoM and the second largest in the world, released an estimated 4.9 million barrels of crude oil (Atlas and Hazen 2011). This significant ecological perturbation of the sea and coastal region has led to negative impacts in local fishing, aquaculture and tourism (National Health Environmental Effects Research Laboratory (US) Gulf Ecology Division 1999; Yanez-Arancibia and Day 2004; Arreguin-Sanchez et al. 2004; Ritchie and Keller 2008). Since the occurrence of the oil spill, most studies used molecular approaches including NextGen sequencing technology to assess the diversity and metabolism of complex microbial communities in oil-contaminated environments, particularly samples collected from offshore locations surrounding the Macondo Blowout (Evans et al. 2004; Bordenave et al. 2007; Cappello et al. 2007; Liang et al. 2007; dos Santos et al. 2011; Kostka et al. 2011; Baelum et al. 2012; Beazley et al. 2012; Kim et al. 2012; Kryachko et al. 2012; Mason et al. 2012; Kimes et al. 2013). Overall, these studies showed that the impacts of oil on GoM indigenous microbial communities were dependent upon the location and the type of samples used. For example, the microbial communities when treated with oil in pristine sediments,  showed an overall decrease in diversity, but the occasional emergence of oil-tolerant and hydrocarbonoclastic bacteria (HCB hereafter) was noticed. However, the communities that were exposed to crude oil released from natural oil-seeps in the GoM ocean floor responded faster than the previously unoiled sediments (Kvenvolden and Cooper 2003). Moreover, the changes in the microbial diversity were found to be dependent upon the complexity of the petroleum hydrocarbon compounds and the length of treatment (Cappello et al. 2007). Although the adverse effects of the Macondo Blowout on the GoM's large open water and sediment ecosystems is indisputable, only a few studies have focused on the effect of MC252 oil on the coastal salt marsh microcosm environment (Beazley et al. 2012; Liu and Liu 2013). The coastal salt marsh along the GoM presents a delicately balanced, highly interactive, dynamic ecosystem that plays a crucial role in nutrient cycling, and serves as an important breeding ground and source of nutrients for coastal flora and fauna, including the Gulf seafood industry (Mitsch and Gosselink 2000; Engle 2011; Silliman et al. 2012; Beazley et al. 2012). Due to the relatively low tidal wave energy level, salt-marsh sediments and plants are capable of trapping and retaining spilt oil for years, often adversely affecting ecosystem structure and function (Mitsch and Gosselink 2000; Engle 2011; Silliman et al. 2012; Beazley et al. 2012). In this study, we evaluated the changes in the indigenous bacterial communities in salt marsh GoM coastal sediment microcosms in vitro following treatment with MC252 oil. We utilized the massively parallel bacterial tag-encoded FLX-amplicon pyrosequencing (bTEFAP) approach, targeting the 16S small subunit (SSU) rRNA to obtain moderate to deep coverage that can also be efficiently processed by bioinformatics pipelines (Sogin et al. 2006; Dowd et al. 2008a, b). This approach has been shown to be effective in assessing the microbial community structure in complex ecosystems with high coverage (Acosta-Martinez et al. 2008; Sundarakrishnan et al. 2012). In addition, we have evaluated the presence of the bacterial biodegradative genes in oil-treated and untreated control sediment metacommunity DNA using the PCR method.

## Materials and methods

### Sample collection and sediment analysis

The sediment and seawater samples from Bayou La Batre, Alabama (N30°22.714′ W088°18.230′), were collected on March 17–31, 2011 in acid-washed plastic containers. The sediments were collected at a single location and placed in a 5 gallon acid-washed plastic bucket. The top 15-30 cm of sediments were sampled 15-30 cm beyond the edge of the salt marsh emergent aquatic macrophytes. The sediment sample was thoroughly homogenized by stirring and used for the microcosm setup. Seawater pH was measured using a combination pH electrode attached to a VWR Symphony SP90M5 meter, which was calibrated with standard buffer solutions to +0.05 pH unit. Seawater alkalinity was determined by titration using SM 2320B (American Water Works Association (AWWA) 2012). The measurements of total dissolved solids (TDS) were made using a METTLER AT261 DeltaRange balance and the gravimetric technique described in Method SM 2540C (AWWA 2012). An aliquot (100 g) of the sediment was crushed using a ceramic-lined SPEX 8510 Shatterbox, and then passed through a 400-mesh sieve. Sediment mineralogy was determined on bulk and oriented clay mounts by X-ray diffraction (XRD) analysis using a Bruker D8 advance diffractometer (Drever 1973). Also total petroleum hydrocarbons and total organic carbon (TOC) were determined by GC–MS using standard methods (Camilli et al. 2010; Pavlova and Papazova 2003; Roling et al. 2004) (Dr. Yuehan Lu, University of Alabama, Tuscaloosa, AL; personal communication) to confirm the initial level of oil, if any, that was present in the sediment samples. For each microcosm setup 326.94 g wet sediment (equivalent to 200 g dry weight each in glass jars) and 173.06 g seawater were placed in a 500 mL glass jar with a Teflon-lined lid. Sterile sediments (autoclaved at 121 °C for 20 min at 15 lb/sq inch pressure) with oil and without oil were used as negative controls. The glass jars were combusted at 450 °C for 5 h prior to their use for the microcosm setup. Mississippi Canyon Block 252 oil (MC252) (50,000 ppm, 5 % by weight) was added to each microcosm, gently mixed  by shaking and incubated at room temperature (20° ± 1 °C) on a shaker table set at 100 rpm to simulate wave action. Non-sterile sediment samples without MC252 oil (T0), used as controls to determine a baseline bacterial community, were also incubated simultaneously. Duplicate sediment samples were analyzed for metagenomics at different time points, time zero (T0, non-sterile untreated sediments), 2 weeks (T2, 2 weeks after the MC252 oil treatment), and 3 weeks (T3, 3 weeks after the MC252 oil treatment). Sediment samples (1 g each in triplicate) from the oil-treated and untreated microcosms (T0, T2 and T3), and oil-treated and -untreated sterile sediments were collected using aseptic techniques and the metacommunity DNA was extracted. High-quality metagenomic DNA was purified in triplicate from each microcosm sample using MoBio PowerSoil^®^ DNA purification kit (MoBio Laboratories Inc., CA; http://www.mobio.com). The concentration (at 260 nm wavelength) and quality (a ratio of 260/280 nm wavelengths = 1. 8 ± 1) of each extracted DNA sample  were determined by a Lambda 2 spectrophotometer (Perkin Elmer, Norwalk, Conn.), followed by agarose gel electrophoresis in Tris–acetate–EDTA buffer (TAE, pH 7.8) (Ausubel et al. 1987). After confirming the purity and concentration, triplicate DNA samples from each oil-treated sediment were pooled and 100 ng of DNA was used by the Research and Testing Laboratories (RTL) (Lubbock, Texas) (http://www.researchandtesting.com) for bacterial tag-encoded FLX-amplicon pyrosequencing (bTEFAP) (Dowd et al. 2008a). For the untreated controls, the same approach for purification and sequencing of DNA was used. The bTEFAP was performed as described previously (Dowd et al. 2008a; Sogin et al. 2006) using primers 341F (5′-CCT ACG GGA GGC AGC AG-3′ (Muyzer et al. 1993) and 907R (5′-CCG TCA ATT CMT TTG AGT TT-3′ (Lane et al. 1985) targeting the variable regions 3 and 5 (V3–V5) of the bacterial 16S rRNA gene (Li et al. 2009). Briefly, the initial generation of the sequencing library was performed by one-step PCR with HotStarTaq Plus Master Mix Kit (Qiagen, Valencia, CA) and 341F and 907R primers. The bTEFAP were conducted on a Roche 454 FLX instrument using the Titanium reagents and the procedure at RTL (Lubbock, TX).

### Sequence processing and bioinformatic workflow

The Quantitative Insights Into Microbial Ecology software package (QIIME, ver.1.8.0) (Caporaso et al. 2010b) was used to analyze the raw barcoded pyrosequence reads from the bacterial 16S rRNA gene. The low-quality sequence reads that did not match the barcode sequences, primer sequences, nucleotide homopolymers longer than 8 bp, ambiguous nucleotides (N), had low-quality scores (quality score <25), or short length of reads (<200 bp) were removed from the library using split_libraries.py command. The sequences were clustered into operational taxonomic units (OTUs hereafter) at 97 % sequence similarity using UCLUST (Edgar 2010). Representative sequences were selected from each OTU, aligned with PyNAST (version 1.1) (Caporaso et al. 2010a) (http://pynast.sourceforge.net/) and taxonomy was assigned to each representative sequence using RDP classifier (version 2.2) (Wang et al. 2007) (http://rdp.cme.msu.edu/) at 50 % confidence. Previous studies have shown that bacterial taxonomy can be accurately predicted at this confidence level (Liu et al. 2008). The aligned representative sequences were then filtered and a phylogenetic tree was generated using FastTree (Price et al. 2009) (http://www.microbesonline.org/fasttree/). An OTU table was constructed through the aforementioned QIIME (ver. 1.8.0) workflow, which was subsequently used to generate the relative proportions of bacterial taxa in these communities.

### Microbial diversity statistics

Further analyses of the bacterial diversity obtained through bTEFAP included estimating alpha and beta-diversity through the QIIME (ver. 1.8.0) software, based on the OTU table. Estimates of alpha-diversity include observed OTUs, Shannon diversity index (Shannon et al. 1964), and Simpson diversity index (Simpson 1949). The Shannon diversity index (Shannon et al. 1964) was defined as $H' = \sum\nolimits_{i = 1}^{s} {(p_{i} \ln p_{i} } )$, where *s* is the number of OTUs in the sample and *p*_*i*_ is the proportion of the organisms in the sample represented by the *i*th OTU. The Simpson diversity index (Simpson 1949) was defined as 1-D, where $D = 1/\sum {p_{i}^{2} }$ and *p*_*i*_ is the proportion of the sample that OTU *i* constitutes.

In order to calculate beta-diversity (differences between samples), a comparison of the bacterial communities in oil-treated and untreated samples was performed using UniFrac metrics (Lozupone et al. 2006) (http://bmf.colorado.edu/unifrac/). Based on the weighted UniFrac phylogenetic distances, a principal coordinate analysis (PCoA) plot and Jackknife statistics were generated by the QIIME pipeline. Furthermore, the OTU table generated by QIIME (ver. 1.8.0) was modified and exported into Cytoscape 2.8.2 (Shannon et al. 2003) (http://www.cytoscape.org/), using the edge-weighted force-directed layout to visualize the data network (Pope et al. 2012). Cytoscape is a software that allows OTU–OTU interactions to be mapped to an OTU network to represent the bacterial similarities and differences between samples.

### PCR amplification of biodegradative genes

The presence of bacterial genes involved in alkane, naphthalene, and catechol biodegradation in oil-treated or untreated control salt-marsh sediment metacommunity DNA were determined by PCR method. Oligonucleotide primers targeting the alkane hydroxylase (*alk*), an ISP (subunit of naphthalene dioxygenase) (*ndoB*), catechol 2,3-dioxygenase (*C2,3DO*), and toluene/biphenyl dioxygenase (*todC1/bphA1*) used in this study for PCR amplification are listed in Table 1. Genomic DNA from *Rhodococcus* sp., *Pseudomonas putida* ATCC 17484, *P. putida* mt2, and *Sphingomonas* 35/1 were used as positive controls for *alkB*, *ndoB*, *C2,3DO*, *todC1*, and *bph* amplification, respectively (Panicker et al. 2010). Each PCR amplification was performed in a 25-µl reaction volume consisting of 1 µg of purified genomic DNA; 200 µM of each of the dNTPs; 1 µM of each of the oligonucleotide primer and 2.0 U Ampli*Taq* (Perkin Elmer, Norwalk, CT) DNA polymerase; and 1× PCR reaction buffer [10× buffer consisted of 300 mM Tris–Cl (pH 9.0), 75 mM (NH_4_)_2_SO_4_, and 2.0 mM MgCl_2_] (Panicker et al. 2010). All PCR amplifications were performed in a GeneAmp PCR system 2400 (Perkin Elmer, Norwalk, CT) thermocycler using the following temperature cycling parameters: initial denaturation at 94 °C for 2 min followed by a total of 30 cycles of amplification in which each cycle consisted of denaturation at 94 °C for 1 min, primer annealing at 60 °C (54 °C for *C2,3DO* and 56 °C for *alkB194*) for 1 min, and primer extension at 72 °C for 2 min (Table 1). After amplification, final extension of the incompletely synthesized DNA was carried out at 72 °C for 7 min (Panicker et al. 2010). The PCR fragments were analyzed by agarose gel electrophoresis (1.5 % wt/vol) using a CloneSizer™ 100 bp DNA ladder (Catalog#: 11600, NORGEN Biotek Corp, Ontario, Canada) as a size standard. The gel was stained with ethidium bromide and visualized under a Photoprep I UV transilluminator (Fotodyne, Inc., Hartland, WI).

**Table 1:** List of oligonucleotide primers, target genes, and other relevant information used in this study for PCR amplification of the presence of the biodegradative genes in the sediment microcosm metacommunity DNA

Primer name	Gene	Primer sequence (5′–3′)	Primer length (nt)	*T*_m_ (°C)^a^	Origin	References
*L*-*alkB*	Alkane hydroxylase	gtatcgtgaacccaactaccgctcaat	27	80	*Pseudomonas oleovorans* ATCC 29347	Kok et al. (1989)
*R*-*alkB*		ggtggaacaccactagatagagacg	25	76		
Rh L-*alkB*1		atctgggcgcgttgggatttgagcg	25	80	*Rhodococcus* sp. strain Q15	Whyte et al. (1998)
Rh R-*alkB*1		cgcatggtgatcgctgtgccgctgc	25	84		
Rh L-*alkB*2		actctggcgcagtcgttttacggcc	25	80	*Rhodococcus* sp. strain Q15	Whyte et al. (1998)
Rh R-*alkB*2		cccactgggcaggttgggcgcaccg	25	88		
Rh L-*alkB*194		cacagytggaacagygatcrc	21	56	*Rhodococcus* sp. strain Q15 degenerate primer to region common to alkb1 and alkb2	Panicker et al. (2010)
Rh R-*alkB*194		tccatcacyttkcgccacag	20	56		
L-*alkB*870G		tggccggctactccgatgatcggaatctgg	30	96	*P. oleovorans* ATCC 29347	Van Beilen et al. (2002)
R-*alkB*870G		cgcgtggtgatccgagtgccgctgaaggtg	30	100		
L-TS2S		aayagagctcaygarytrggtcayaag	27	60	*P. oleovorans* GPo1 and *Acinetobacter* sp. ADP1	Phillips et al. (2000)
R-deg1RE		gtragictrgtrgtrcgcttaaggtg	26	64		
(Ac) *alkM*-F		cctgtctcatttggcgctcgttcctacagg	30	94	*Acinetobacter* sp. ADP-1	Ratajczak et al. (1998)
(Ac) *alkM*-R		ccaaagtggcggaatcatagcaggc	25	78		
L-*ndoB*	Naphthalene dioxygenase	cactcatgatagcctgattcctgaccccggcg	32	102	*Pseudomonas putida* ATCC 17484	Kurkela et al. (1988)
R-*ndoB*		ccgtcccacaacacacccatgccgctgccg	30	102		
L-*cat238*	Catechol 2,3 dioxygenase	cgacctgatctccatgaccga	21	66	Degenerate primer from conserved region of C23DO gene in *Pseudomonas* sp.	Mesarch et al. (2000)
R-*cat238*		tcaggtcagcacggtca	17	54		
*xylE*b-F		gtgcagctgcgtgtactggacatgagcaag	30	94	*Pseudomonas putida* ATCC 33015	Nakai et al. (1983)
*xylE*b-R		gcccagctggtcggtggtccaggtcaccgg	30	104		
*cat2,3* 1a-F		aggtgctcggtttctacctggccg	24	78	*Pseudomonas putida* ATCC 33015	Laramee et al. (2000)
*cat2,3* 6a-R		acggtcatgaatcgttcgttgag	23	68		
*todC*1-F	Toluene dioxygenase	cgggtgggcttacgacaccgccggcaatct	30	100	*Pseudomonas putida* mt2	Panicker et al. (2010)
*todC*1-R		tcgagccgcgctccacgctacccagacgtt	30	100		
*bphA*1-F	Biphenyl dioxygenase	tcacctgcagctatcacggctgg	23	74	*Sphingomonas* 35/1	Panicker et al. (2010)
*bphA*1-R		ggatctccacccagttctcgccatcgtcctg	31	100		

^a^ *T*_m_ (°C) = 2(A + T) + 4(G + C) (Ausubel et al. 1987)

## Results

### Sea water and sediment samples

The seawater pH was 7.66, alkalinity = 83.07 mg CaCO_3_/L, and total dissolved solids = 2,916 mg/L (brackish). The sediment mineralogy was largely quartz, with trace amounts of kaolinite, saponite, microcline and hematite. Sediment total petroleum hydrocarbon (TPH) concentration was below detection level in the original untreated sediment; and the sediment total organic carbon (TOC) ranged from 0.88–1.45 % (Dr. Yuehan Lu, University of Alabama, Tuscaloosa, AL).

### Total pyrosequencing reads and OTUs

A total of 8,172 partial (V3–V5) 16S rRNA “raw” sequence reads were generated by bTEFAP from the three microcosm samples. After stringent quality-based trimming, 5,264 quality reads were used for further bioinformatics analysis. Within these reads, 1,542 reads were clustered into 498 distinct OTUs (at 97 % sequence similarity) from the T0 sample, 2,003 reads (908 OTUs at 97 % sequence similarity) from the T2 sample, and 1,719 reads (282 OTUs at 97 % sequence similarity) from the T3 sample were achieved. No quality pyrosequence reads were observed from the sterile negative control sediment samples.

### Taxonomy-based bacterial diversity

**Fig. 1:**
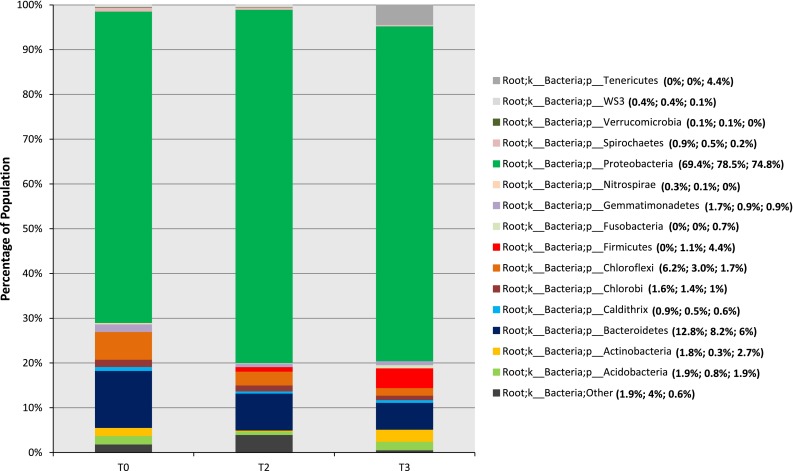
Stacked column bar graph showing the relative abundance of the phyla distribution within the three microcosm samples collected from Bayou La Batre of coastal Alabama of the Gulf of Mexico. The percentage of each phylum is shown on the *right side* of the legend with the numbers corresponding to the 0 h non-sterile untreated control (T0), 2-week oil-treated (T2), and 3-week oil-treated (T3) samples

The overall indigenous bacterial taxonomy represented by the Bayou La Batre, Alabama, salt-marsh sediment microcosm in vitro (oil-treated and untreated) was distributed amongst 15 defined phyla, 37 genera, and a few undefined taxa (referred to as “other” in this manuscript) (Fig. 1; Supplementary Table 1). Overall, the relative abundance of the bacterial communities in the 2- and 3-week untreated control samples were not significantly different when compared with the T0 sample; therefore, only the T0 data are presented in this manuscript (Fig. 1). Similar observations were reported in untreated water samples (0 h, 5 days, and 20 days) from the Gulf of Mexico and mangrove sediment samples (0 h and 23 days) from Rio de Janeiro, Brazil (dos Santos et al. 2011; Baelum et al. 2012). The largest number of bacterial sequences identified in untreated sediments belonged to the phylum Proteobacteria and which, as compared to the T0 sample (at 69.4 % of total bacterial load), increased in the T3 sample (74.8 %) (Fig. 1). Besides phylum Proteobacteria, phylum Firmicutes exhibited an overall increase in the T3 sample from undetectable to 4.4 %. Similarly, phyla Tenericutes and Actinobacteria increased from undetectable to 4.4, and 1.8 to 2.7 %, respectively, as compared to the T0 sample (Fig. 1). A decreasing trend of 2–4 times in the T3 sample was noticed for bacteria representing phyla Spirochaetes, Chloroflexi, WS3, Bacteriodetes, Nitrospira, Gemmatimonadetes, Chlorobi, and Cladithrix, whereas phyla Acidobacteria and Fusobacteria remained virtually unchanged (Fig. 1).

**Fig. 2:**
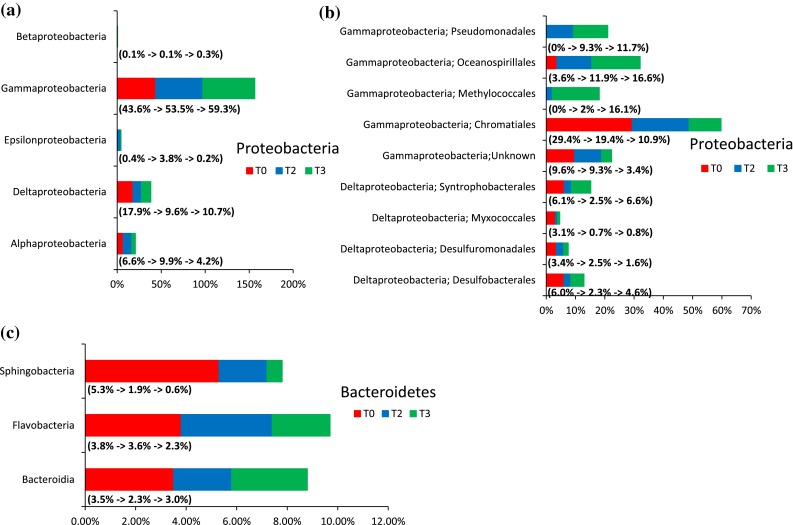
Comparison of the three salt-marsh sediment samples showing the taxa at the order level in the non-sterile untreated (T0) sample and those treated with MC252 oil. **a** Distribution of class within phylum Proteobacteria; **b** distribution of order within phylum Proteobacteria; **c** distribution of class within phylum Bacteroidetes. The percentage of each class and order level are shown on the *right side* of the legend with the numbers corresponding to the 0 h non-sterile untreated control (T0), 2-week oil-treated (T2), and 3-week oil-treated (T3) samples

**Fig. 3:**
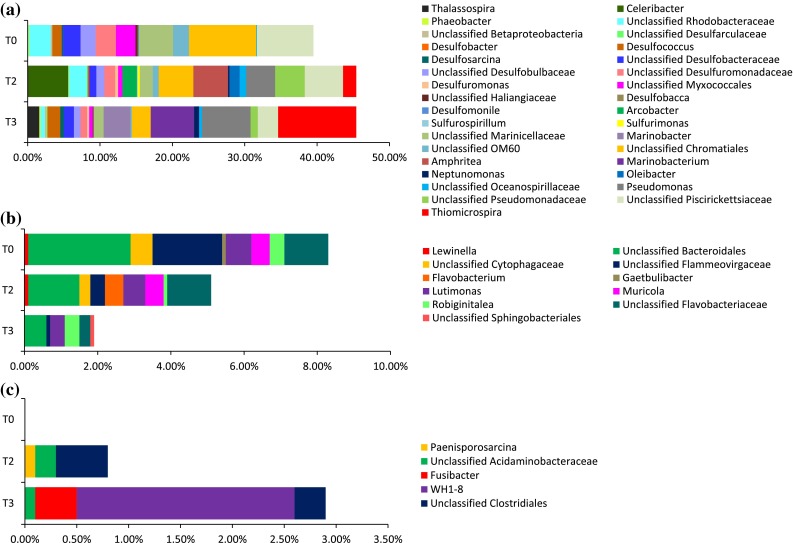
Stacked column bar graph showing the relative abundances of genera in the 0 h non-sterile untreated control (T0), 2-week oil-treated (T2) and 3-week oil-treated (T3) microcosm samples. The figure shows the genus level distribution within **a** phylum Proteobacteria; **b** phylum Bacteroidetes; and **c** phylum Firmicutes

 Because members of the phylum Proteobacteria dominated the bacterial community, we further analyzed the taxa within this phylum at the class (Fig. 2a) and genus levels (Fig. 3a). In the T0 sample, class Gammaproteobacteria constituted ~43.6 % of the total population, followed by Deltaproteobacteria (17.9 %). In the T3 sample, the microbial composition in Gammaproteobacteria increased to an approximately 59.3 %; and Deltaproteobacteria decreased to 10.7 % (Fig. 2a). Furthermore, the classes Alphaproteobacteria and Epsilonproteobacteria first increased their relative abundances in the T2 and then decreased in the T3 sample (Fig. 2a). No significant change was noticed in Betaproteobacteria over the 21 day incubation in all three samples (Fig. 2a).

Further analysis of the members of Gammaproteobacteria determined that order Chromatiales (29.4 %) dominated the bacterial load followed by Oceanospirillales (3.6 %) in the T0 sample (Fig. 2b). In the T3 sample, order Chromatiales decreased to 10.9 %, whereas order Oceanospirillales increased from 3.6 to 16.6 %, Pseudomonadales from undetectable to 11.7 %, Methylococcales from undetectable to 16.1 % (Fig. 2b). At the genus level within Gammaproteobacteria, *Neptunomonas*, *Pseudomonas*, and unclassified *Oceanospirillaceae* were increased in the T2 sample and maintained their population in the T3 sample (Fig. 3a). *Oleibacter*, *Amphritea*, and unclassified *Pseudomonadaceae* were increased in the T2 sample, but returned to the basal level in the T3 sample (Fig. 3a). *Marinobacter*, *Thiomicrospira*, and *Marinobacterium* were increased only in the T3 sample (Fig. 3a). Other genera, such as unclassified *Marinicellaceae*, OM60, *Chromatiales*, and *Piscirickettsiaceae* decreased in abundance in both the T2 and T3 samples (Fig. 3a). Class Deltaproteobacteria was dominated by order Syntrophobacterales (6.1 %) followed by the orders Desulfobacterales (6.0 %) (a group exclusively belonging to the sulfur-reducing bacteria), Desulfuromonadales (3.4 %), and Myxococcales (3.1 %) in the T0 sample (Fig. 2b). There was a decreasing trend in the relative abundance within different orders of the class Deltaproteobacteria after oil treatment, except for a slight increase in both orders Syntrophobacterales, and Desulfobacterales at 21 days (T3) (Fig. 2b). At the genus level, *Desulfuromonas* and unclassified *Desulfarculaceae* increased in the T2 sample and maintained their population in the T3 samples. Genera *Desulfobacter*, *Desulfococcus*, and *Desulfosarcina*, increased in the T3 sample (Fig. 3a). Other genera, *Desulfobacca*, *Desulfomonile*, and members within families unclassified *Desulfobacteraceae*, *Desulfobulbaceae*, *Desulfuromonadaceae*, *Myxococcales*, and *Haliangiaceae,* decreased in relative abundance after the oil treatment (Fig. 3a).

Other genera, *Thalassospira*, *Celeribacter*, *Phaeobacter*, and members of the family  unclassified Rhodobacteraceae were found within class Alphaproteobacteria (Fig. 3a). The relative abundance of genus *Celeribacter* increased in both T2 and T3 while genus *Thalassospira* increased only in T3 (Fig. 3a). However, *Phaeobacter* and unclassified *Rhodobacteraceae* decreased in relative abundances after oil treatment (Fig. 3a). Within class Betaproteobacteria, we were able to identify unclassified *Betaproteobacteria*, but their relative abundance remained unchanged after the oil treatment (Fig. 3a). Genera *Arcobacter*, *Sulfurospirillum*, and *Sulfurimonas*, within the class Epsilonproteobacteria were detected and increased their abundance in the T2 sample first, then returned to the basal level in the T3 sample (Fig. 3a).

Because members of the phylum Bacteroidetes are the second most abundant phylum in all three samples, we further analyzed the taxa within this phylum at the class level (Fig. 2c). Class Sphingobacteria dominated the bacterial load, followed by Flavobacteria and Bacteroidia in the T0 sample (Fig. 2c). However, in the T3 sample, the relative abundances of Sphingobacteria and Flavobacteria decreased (Fig. 2c). Although class Bacteriodia slightly decreased in the T2 sample, remained unchanged overall, comparing to the initial T0 number to that of the T3 sample (Fig. 2c). Most genera, *Lewinella*, *Gaetbulibacter*, *Lutimonas*, *Muricola*, unclassified *Bacteroidales*, *Cytophagaceae*, *Flammeovirgaceae*, and *Flavobacteriaceae,* within phylum Bacteroidetes decreased in population after the oil treatment, except *Flavobacterium*, *Robiginitalea*, and unclassified *Sphingobacteriales* (Fig. 3b). *Flavobacterium* increased their abundance only in the T2 sample, and *Robiginitalea* and unclassified *Sphingobacteriales* increased their population only in the T3 sample (Fig. 3b).

We also detected genera *Paenisporosarcina*, *Fusibacter*, *WH1*-*8*, unclassified *Acidaminobacteraceae* and *Clostridiales* within phylum Firmicutes; all genera increased their abundance in sediments treated with oil (T2 and T3) (Fig. 3c). *Paenisporosarcina,* unclassified *Acidaminobacteraceae,* and *Clostridiales* increased in their relative abundance in the T2 sample and returned to the basal level in the T3 sample (Fig. 3c). However, *Fusibacter* and *WH1*-8 increased their relative abundance only in the T3 sample (Fig. 3c).

### Statistics-based bacterial diversity

**Fig. 4:**
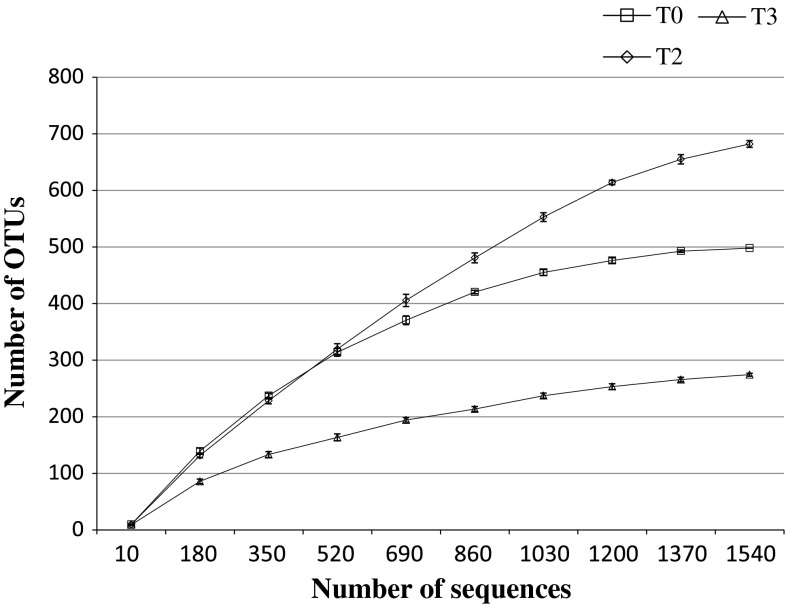
Rarefaction curves based upon bacterial 16S rRNA genes generated after normalizing the data for the number of sequences obtained from the 0 h non-sterile untreated control (T0), 2-week (T2) and 3-week (T3) oil-treated microcosm samples. The number of unique OTUs at 3 % sequence variations and standard deviation  was calculated by QIIME (ver. 1.8.0)

The Shannon diversity (Shannon et al. 1964) and Simpson diversity (Simpson 1949) indices exhibited a slight decrease in bacterial diversity in the oil-treated sediment sample (Table 2). Rarefaction curves indicated that total quality sequences from each of the microcosm experiments were approaching saturation when constructed at a 3 % sequence variation (Fig. 4).

**Table 2:** Number of sequences and OTUs, Shannon diversity and Simpson diversity indices, calculated based upon bacterial 16S rRNA gene sequences in the datasets of the 0 h non-sterile untreated control (T0), 2-week oil-treated (T2), and 3-week oil-treated (T3) microcosm samples, determined using QIIME (ver. 1.8.0)

Samples	Number of sequences	Number of OTUs	Shannon diversity index	Simpson diversity index
T0	1,542	498	8.474	0.995
T2	2,003	908	8.425	0.99
T3	1,719	282	6.443	0.964

**Fig. 5:**
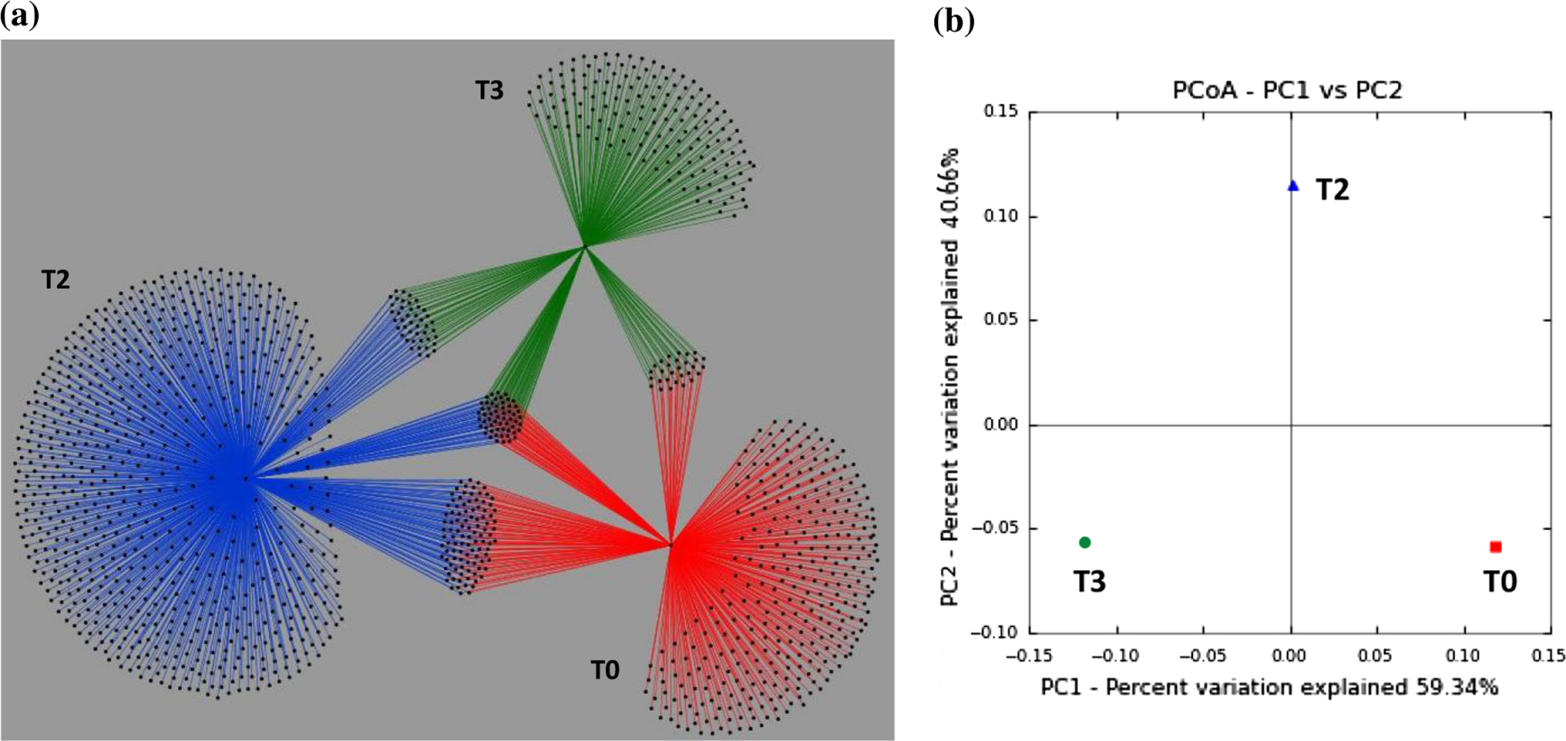
Operational taxonomic unit (OTU) network and PCoA (principle coordinate analysis) plot. **a** OTU network analysis to evaluate the effect of oil treatment through the relationship between OTUs (each *dot*) in the three different microcosm samples. Edges of the 0 h non-sterile untreated control sample (T0) are represented in *red*, the 2-week oil-treated sample (T2) in *blue*, and the 3-week oil-treated sample (T3) in *green.* **b** The PCoA plot based on weighted UniFrac shows the differences and similarities between the bacterial communities from each microcosm experiment

The relationships between the bacterial community structures among the three samples were further analyzed through the creation of an OTU network, and statistically compared using the weighted UniFrac. In the OTU network (Fig. 5a), each dot represents an OTU and each edge represents different sediment samples (T0 in red, T2 in blue, and T3 in green). The OTU network clearly shows that each of the microcosm samples has their own unique bacterial community, but many OTUs are shared between the T0 and T2 samples (Fig. 5a). Furthermore, the PCoA plot demonstrated that there were significant differences in the bacterial community structure between the three samples (Fig. 5b). These results indicated that the oil treatment resulted in shifting the microbial community structure over the course of the experiment.

### Biodegradative genes

The PCR amplification targeting the biodegradative genes in purified metacommunity DNA from oil-treated sediments exhibited positive detection of alkane hydroxylase (*alkB*), catechol 2,3-dioxygenase (*C2,3DO*), and biphenyl dioxygenase (*bph*) biodegradative genes (Table 3). Positive amplification of the biphenyl dioxygenase (*bph*) gene in the T0 sample indicates that HCB exist in the GoM salt-marsh sediment, and were likely enriched following oil treatment due to a shift of the indigenous bacterial population.

**Table 3:** Presence of biodegradative genes in the microcosm metacommunity DNA extracted from the 0 h non-sterile untreated control (T0) and 2-week (T2) and 3-week (T3) oil-treated microcosm samples

Sample I.D. detail description	Alkane hydroxylase (*all*)							Naphthalene dioxygenase (*ndoB*)	Catechol 2,3 dioxygenase (*C2,3DO*)			Toluene dioxygenase (*tod*)	Biphenyl dioxygenase (*bph*)
	*alkB* (546 bp)	Rh-*alkB*1 (629 bp)	Rh-*alkB*2 (552 bp)	Rh-*alkB*194 (194 bp)	*alkB*870G (870 bp)	TS2S/deg1RE (550 bp)	(Ac) *alkM* (496 bp)	*ndoB* (642 bp)	*C2,3DO* (238 bp)	*XylEb* (834 bp)	*Cat2,31a* (405–408 bp)	*todC1* (560 bp)	*bphA1* (830 bp)
T0	−	−	−	−	−	−	−	−	−	−	−	−	+
T2	−	−	−	−	+	−	−	−	+	−	+	−	+
T3	−	−	−	−	+	−	−	−	+	−	+	−	+

+ = PCR positive detection; − = PCR negative detection

## Discussion

The application of bTEFAP and downstream bioinformatic analyses on the metacommunity DNA indicated that the salt-marsh sediments of Bayou La Batre in coastal Alabama contain a diverse bacterial community with phylum Proteobacteria, as the dominant taxa. Similar result was previously reported for mangrove and salt-marsh sediments by studies using molecular approaches, including the 454 pyrosequencing (Liang et al. 2007; dos Santos et al. 2011; Beazley et al. 2012; Liu and Liu 2013). Although the results from this study show that the oil treatment altered the overall bacterial communities, it is important to identify how these communities differed in the treated and the untreated control sample at various taxa levels. To achieve this, we first identified the OTUs, followed by monitoring significant changes in their relative abundances in the oil-treated samples (Figs. 1, 2, 3). Based upon these analyses, we were able to categorize three distinct groups of bacteria: (1) early responders, which increased their relative abundance by the second week of treatment (T2); (2) early, transient responders, which increased in relative abundance by the second week (T2), but returned to the basal level (i.e., that of the untreated sediment) by the third week of oil treatment (T3); and (3) late responders, which increased only after 3 weeks of oil treatment (T3).

The early responders, which belonged to the class Gammaproteobacteria and order Pseudomonadales, were only detected in the oil-treated samples. At the genus level, we detected *Pseudomonas* as an early responder (Fig. 3a). It has been reported that bacteria belonging to order Pseudomonadales are capable of biodegradation of alkanes and naphthalene by using genetic systems present on the OCT-plasmid (Kok et al. 1989; van Beilen et al. 1994). Detection of this genus in our study was supported by the PCR-positive results of alkane and catechol degradation genes (Table 3), and an increase in their abundance in both the T2 and T3 samples (Fig. 2b). Another early responder, order Oceanospirillales (class Gammaproteobacteria), increased rapidly in the T2 sample, and then continued to increase, but at a slower rate, in the T3 sample (Fig. 2b). At the genus level, we found *Neptunomonas* and unclassified *Oceanospirillaceae* as early responders (Fig. 3a). Previous studies showed that *Oceanospirillales* proliferate rapidly in oil-enriched environments and are capable of mineralizing *n*-alkane and cycloalkane (Hazen et al. 2010; dos Santos et al. 2011; Redmond and Valentine 2012; Mason et al. 2012; Baelum et al. 2012; Chakraborty et al. 2012). Also, previous study has supported hydrocarbon degradation activity by genus *Neptunomonas* (Hedlund et al. 1999).

Among the transient responders, we identified order Rhodobacterales belonging to the class Alphaproteobacteria, which increased in the T2 sample and then decreased in the T3 sample (data not shown). At the genus level, *Celeribacter* was assigned as transient responders and they increased their abundance after the oil treatment (Fig. 3a). Previously, members of the order Rhodobacterales were detected in oil-enriched environments and described to have the ability to degrade hydrocarbon compounds (Brakstad and Lodeng 2005; Hernandez-Raquet et al. 2006; Chakraborty et al. 2012; Redmond and Valentine 2012; Baelum et al. 2012). The genera *Amphritea*, *Oleibacter*, and unclassified Pseudomonadaceae belonging to the class Gammaproteobacteria also increased their relative abundance as transient responders in the T2 sample. It has been reported in a previous study that although members of the class Gammaproteobacteria although steadily increased up to 26 days following oil treatment, they were replaced by Alphaproteobacteria over time. This is perhaps due to the ability of the Alphaproteobacteria to utilize residual PAHs or the byproducts of alkanes (Roling et al. 2002). Similarly, we found that the abundance of Gammaproteobacteria steadily increased in the T2 and T3 samples (Sette et al. 2007). Interestingly, the abundance of Alphaproteobacteria (genus *Celeribacter*) and Epsilonproteobacteria (genera *Arcobacter*, *Sulfurospirillum*, and *Sulfurimonas*) increased in the T2 sample, but unlike other studies, decreased in the T3 sample (Figs. 2a, 3a). It has been reported that members of the class Epsilonproteobacteria are the key players in nitrogen and sulfur cycling in marine environments and are capable of withstanding hydrocarbons in contaminated environments (Wirsen et al. 2002; Grabowski et al. 2005; Campbell et al. 2006; Gupta 2006; Sette et al. 2007; Prabagaran et al. 2007; Herrmann et al. 2010; Hubert et al. 2012). The reason for the decrease in the relative abundance of Alphaproteobacteria, especially genera *Phaeobacter* and unclassified *Rhodobacteraceae* in the T3 sample remains unclear. Other genera such as *Flavobacterium* were assigned as transient responders and their hydrocarbon degrading activity has been reported in a previous study (Hemalatha and Veeramanikandan 2011).

The late responders belonged primarily to the order Clostridiales within phylum Firmicutes, which have been known to degrade hydrocarbons and to be an indicator bacteria in oil-contaminated environments (Howarth 1984; Bordenave et al. 2007; dos Santos et al. 2011; Mason et al. 2012). These were not detected in the untreated control sediment, but increased rapidly in the T3 sample (data not shown). Within phylum Firmicutes, genera *Fusibacter* and *WH1*-*8* were late responders; *Fusibacter,* especially, has been reported to degrade crude oil (Hasegawa et al. 2014). The members of class Mollicutes within the phylum Tenericutes also appeared as late responders in our study and only detected in the T3 sample (data not shown). In addition, Methylococcales from class Gammaproteobacteria showed a significant increase in the T3 sample (Fig. 2b), which is similar to previous reports that this bacteria increased in their relative abundance in oil-treated environments and bloomed slowly, compared to other bacteria (Mason et al. 2012; Chakraborty et al. 2012; Redmond and Valentine 2012; Baelum et al. 2012). Within the phylum Proteobacteria, genera such as *Thalassospira*, *Desulfobacter*, *Desulfococcus*, *Desulfosarcina*, *Marinobacter*, *Marinobacterium*, and *Thiomicrospira* were late responders because they increased their relative abundance in the T3 sample. According to previous studies, genera *Desulfobacter*, *Desulfococcus,* and *Desulfosarcina* are known to be sulfate-reducing bacteria (Elshahed et al. 2003), and *Marinobacter*, *Marinobacterium,* and *Thalassospira* show hydrocarbon degrading activity of crude oil (Deppe et al. 2005; Yakimov et al. 2007; Kostka et al. 2011). Also, within the phylum Bacteroidetes, genera *Robiginitalea*, and unclassified *Sphingobacteriales* were late responders and known as hydrocarbon degrading bacteria (Kostka et al. 2011).

Further, as reported for the mangrove ecosystems in Brazil (Cappello et al. 2007), we identified members from order Chromatiales (within class Gammaproteobacteria) and order Syntrophobacterales (within class Deltaproteobacteria) to be sensitive to oil treatment, as shown by their decreased relative abundance in the T2 sample (Fig. 2b).

Order Chromatiales within class Gammaproteobacteria consisted of family Chromatiaceae and Ectothiorhodospiraceae—a group of sulfur-oxidizing bacteria that exhibited a steadily decreasing trend in oil-treated sediments. Similarly, class Deltaproteobacteria dominated by the order Syntrophobacterales, consisting of anaerobic sulfate-reducing bacteria, also decreased in the T2 sample but increased in the T3 sample. It has long been established that salt-marsh sediments have high levels of sulfur from the sea water and abundant sulfur oxidizing and sulfate-reducing bacteria found in these ecosystems, and they contribute to the sulfur biogeochemical cycle (Howarth 1984; Teal 1986; Devereux et al. 1996; Glória Pereira et al. 2002; Klepac-Ceraj et al. 2004). Therefore, results of our bTEFAP analysis can likely be explained by the high sulfur content of the salt-marsh sediment and local microaerophilic conditions induced by the oil, which increased a selective group of sulfur-oxidizers, leading to increased sulfur oxidation. This was in concert with the simultaneous decrease in the sulfur reducing group of class Deltaproteobacteria in the T2 sample. But after 2 weeks, due to the intense activities of sulfur-oxidizing bacteria, we suspect that there was depletion of oxygen and severe acidification, leading to an increase in the sulfur-reducing bacteria belonging to the class Deltaproteobacteria. Sulfate-reducing bacteria (class Deltaproteobacteria) have often been found in marine sediments associated with hydrocarbons (Teal 1986; Phelps et al. 1998; Roling et al. 2004; Kimes et al. 2013). The microaerophilic or anoxic conditions of the treated sediment samples were also indicated by the presence of sequences affiliated with an anaerobic group of bacteria mentioned above, and were not present in the untreated sample.

For the bTEFAP metagenomic sequence library, the plateau in a rarefaction curve (Hughes and Hellmann 2005) suggests that the ecosystem has been sampled to saturation with respect to species diversity (Fig. 4). The weighted UniFrac test that accounts for changes in the relative abundances of lineages between communities (Lozupone et al. 2007) showed that bacterial taxa in the T0 sample have greater similarity to those in the T2 sample than to those in the T3 sample (Fig. 5b). This result suggests that the typically undetectable oil-tolerant HCB proliferated shortly after oil treatment and were therefore detected in the T2 sample. We analyzed the microbial response to MC252 oil following 2- and 3-week treatment, because it has been reported that the HCB population is established within 2 weeks as a response to rapid biodegradation events (Head et al. 2006; Yakimov et al. 2007). Overall, the richness of the microbial communities in the treated sediments increased in the T2 sample and then decreased in the T3 sample (Table 2). This implies that perhaps the indigenous HCB flourished, as their presence in the GoM ecosystem, due to the natural seepage of oil or oil-related anthropogenic activities. The decrease in bacterial richness and diversity in the T3 sample was probably due to the growth-limiting toxic effect of polyaromatic hydrocarbons on the oil-sensitive bacteria in the sediments. Furthermore, in this study, PCR amplification of a suite of signature petroleum hydrocarbon degrading genes from oil-treated and untreated sediment metacommunity DNA revealed the presence of alkane and catechol degradation genes only in oil-treated salt-marsh sediments. The presence of these genes in the proliferating hydrocarbon-degrading HCB, particularly in the T2 and T3 samples, supports the availability of biodegradable alkanes, biphenyl and other aromatic hydrocarbons as substrates in the MC252 oil (Mason et al. 2012). Moreover, bacterial taxa capable of actively utilizing the aforementioned hydrocarbon compounds have been described in the previous section.

The present investigation of the indigenous microbial communities of salt-marsh sediments from coastal Alabama and assessment of the impact of oil treatment conducted in vitro utilizing sediment-seawater microcosms, identified the presence of a highly diverse, adaptable, and sustainable microbial community capable of shifting its population dynamics to combat ecological perturbations. These results also revealed the capability and efficiency of targeted metagenomics (bTEFAP) technology (Sogin et al. 2006; Dowd et al. 2008a) in detecting rare groups by providing a fine-resolution map of microbial community structure at different taxonomic levels.

## Electronic supplementary material

Below is the link to the electronic supplementary material.

Supplementary material 1 (DOCX 19 kb)
