# Supplementary material for: Bacterial community shift in the coastal Gulf of Mexico salt-marsh sediment microcosm in vitro following exposure to the Mississippi Canyon Block 252 oil (MC252)
Source: 3 Biotech. 2014 Jul 10;5(4):379–92. doi: 10.1007/s13205-014-0233-x (PMC4522729; doi:10.1007/s13205-014-0233-x)
Supplement: Supplementary file 1 — Supplementary material 1 (DOCX 19 kb) [file 13205_2014_233_MOESM1_ESM.docx]

Bacterial community shift in the coastal Gulf of Mexico salt-marsh sediment microcosm *in vitro* following exposure to the Mississippi Canyon Block 252 oil (MC252)

Hyunmin Koo^1^, Nazia Mojib^1,3^, Jonathan P. Huang^1^, Rona J. Donahoe^2^ and Asim K. Bej^1*^

^1^Department of Biology, University of Alabama at Birmingham, Birmingham, AL 35204

^2^Department of Geological Sciences, University of Alabama, Tuscaloosa, AL 35487

^3^ Current Address: Red Sea Research Center, King Abdullah University of Science and Technology (KAUST), Thuwal, Saudi Arabia.

Supplementary Table 1. The relative abundance of different taxa (up to genus level) classified from the Bayou La Batre, Alabama, through RDP Classifier (at 50% confidence) within the QIIME (ver. 1.8.0) bioinformatics pipeline.

| **Taxon** | T0 | T2 | T3 |
| --- | --- | --- | --- |
| **Phylum Acidobacteria** | + | + | + |
| **Class: OS-K** | + | - | + |
| **Class: RB25** | + | + | + |
| **Class: Sva0725** | + | - | + |
| **Phylum Actinobacteria** | + | + | + |
| **Class Acidimicrobiia** | + | + | + |
| **Phylum Bacteroidetes** | + | + | + |
| **Class Bacteroidia** | + | + | + |
| **Family Marinilabiaceae** | + | - | - |
| **Family SB-1** | + | + | + |
| **Family VC21_Bac22** | - | + | - |
| **Class Saprospirae** | + | + | + |
| **Family Saprospiraceae** | + | + | + |
| *Lewinella* | + | + | - |
| **Class Cytophagia** | + | + | + |
| **Family Cytophagaceae** | + | - | - |
| **Family Flammeovirgaceae** | + | + | + |
| **Class Flavobacteriia** | + | + | + |
| **Family Flavobacteriaceae** | + | + | + |
| *Flavobacterium* | - | + | - |
| *Gaetbulibacter* | + | - | - |
| *Lutimonas* | + | + | + |
| *Muricola* | + | + | - |
| *Robiginitalea* | + | + | + |
| Other | + | + | + |
| **Class Sphingobacteriia** | - | - | + |
| **Phylum Caldithrix** | + | + | + |
| **Class Caldithrixae** | + | + | + |
| **Family Caldithrixaceae** | + | + | + |
| *LCP-26* | + | + | + |
| **Family BA059** | + | + | + |
| **Phylum Chlorobi** | + | + | + |
| **Class Ignavibacteria** | + | + | + |
| **Family Ignavibacteriaceae** | + | + | + |
| **Phylum Chloroflexi** | + | + | + |
| **Class Anaerolineae** | + | + | + |
| **Class Dehalococcoidetes** | + | + | - |
| **Phylum Firmicutes** | - | + | + |
| **Class Bacilli** | - | + | - |
| **Family Planococcaceae** | - | + | - |
| *Paenisporosarcina* | - | + | - |
| **Class Clostridia** | - | + | + |
| **Family Acidaminobacteraceae** | - | + | + |
| *Fusibacter* | - | - | + |
| *WH1-8* | - | - | + |
| Other | - | + | + |
| **Phylum Fusobacteria** | - | - | + |
| **Class Fusobacteriia** | - | - | + |
| **Family Fusobacteriaceae** | - | - | + |
| *Propionigenium* | - | - | + |
| *Psychrilyobacter* | - | - | + |
| **Phylum Gemmatimonadetes** | + | + | + |
| **Class Gemm-1** | + | - | - |
| **Class Gemm-2** | + | + | + |
| **Class Gemm-4** | + | + | + |
| **Class Gemmatimonadetes** | + | - | - |
| **Family Gemmatimonadaceae** | + | - | - |
| *Gemmatimonas* | + | - | - |
| **Phylum Nitrospirae** | + | + | - |
| **Class Nitrospira** | + | + | - |
| **Family Thermodesulfovibrionaceae** | + | + | - |
| **Phylum Proteobacteria** | + | + | + |
| **Class Alphaproteobacteria** | + | + | + |
| **Familly Kiloniellaceae** | - | + | + |
| *Thalassospira* | - | + | + |
| **Familly Cohaesibacteraceae** | + | - | - |
| **Familly Hyphomicrobiaceae** | + | + | + |
| **Familly Phyllobacteriaceae** | + | + | - |
| **Familly Rhodobacteraceae** | + | + | + |
| *Celeribacter* | - | + | - |
| *Citreimonas* | - | + | - |
| *Phaeobacter* | + | + | + |
| *Rhodobacter* | + | - | - |
| **Familly Rhodospirillaceae** | + | + | - |
| *magnetite-containing* | + | - | - |
| **Familly Erythrobacteraceae** | + | - | - |
| **Class Betaproteobacteria** | + | + | + |
| **Class Deltaproteobacteria** | + | + | + |
| **Family Desulfobacteraceae** | + | + | + |
| *Desulfobacter* | - | - | + |
| *Desulfococcus* | + | + | + |
| *Desulfosarcina* | + | - | + |
| Other | + | + | - |
| **Family Desulfobulbaceae** | + | + | + |
| **Family Desulfuromonadaceae** | + | + | + |
| *Desulfuromonas* | - | + | + |
| **Family Haliangiaceae** | + | - | + |
| **Family Syntrophaceae** | + | - | - |
| *Desulfobacca* | + | - | - |
| *Desulfomonile* | + | - | - |
| **Class Epsilonproteobacteria** | + | + | + |
| **Family Campylobacteraceae** | - | + | - |
| *Arcobacter* | - | + | - |
| *Sulfurospirillum* | - | + | - |
| **Family Helicobacteraceae** | + | + | - |
| *Sulfurimonas* | - | + | - |
| **Class Gammaproteobacteria** | + | + | + |
| **Family Marinicellaceae** | + | + | + |
| **Family Alteromonadaceae** | - | - | + |
| *Marinobacter* | - | - | + |
| **Family OM60** | + | + | + |
| **Family Ectothiorhodospiraceae** | + | - | - |
| **Family Oceanospirillaceae** | - | + | + |
| *Amphritea* | - | + | - |
| *Marinobacterium* | - | - | + |
| *Marinomonas* | - | + | - |
| *Neptunomonas* | - | + | + |
| *Oleibacter* | - | + | - |
| Other | - | + | + |
| **Family Pseudomonadaceae** | - | + | + |
| *Pseudomonas* | - | + | + |
| **Family Piscirickettsiaceae** | + | + | + |
| *Thiomicrospira* | - | + | + |
| **Phylum Spirochaetes** | + | + | + |
| **Class Spirochaetes** | + | + | + |
| **Family Spirochaetaceae** | + | + | + |
| *Spirochaeta* | + | + | - |
| **Phylum Tenericutes** | - | - | + |
| **Class Mollicutes** | - | - | + |
| **Phylum Verrucomicrobia** | + | + | - |
| **Class Opitutae** | + | - | - |
| **Phylum WS3** | + | + | + |
| **Class PRR-12** | + | + | + |

^1^(T0) = Non-oil treated; ^2^(T2,T3) = Oil-treated
